# Burden and frequency of viral testing of kidney and non-kidney transplant recipients

**DOI:** 10.1128/spectrum.03575-23

**Published:** 2024-05-06

**Authors:** Hema Kapoor, Caixia Bi, Martin H. Kroll, Ann E. Salm, Edward A. Dominguez

**Affiliations:** 1 Infectious Diseases/Immunology, Quest Diagnostics, Secaucus, New Jersey, USA; 2 Medical Informatics, Biostatistics and Outcomes Research Group, Quest Diagnostics, Secaucus, New Jersey, USA; 3 Analytics and Statistical Applications, Quest Diagnostics, Secaucus, New Jersey, USA; 4 Organ Transplant Infectious Disease, Methodist Transplant Specialists, Dallas, Texas, USA; Montefiore Medical Center and Albert Einstein College of Medicine, Bronx, New York, USA

**Keywords:** post-transplant, infections, molecular, kidney, transplant, infectious disease

## Abstract

Transplant patients are at risk of infections due to long-term immunosuppression contributing to morbidity and mortality in this population. Post-transplant testing guidelines were established to monitor and guide therapeutic interventions in transplant recipients. We hypothesize that there are gaps in adherence to the recommended frequency of laboratory testing in post-transplant patients. We analyzed national reference laboratory data to compare viral post-transplant infection (PTI) testing frequency with their respective published guidelines to understand patient uptake and compliance. We evaluated the ordering patterns, positivity rates, and frequency of molecular infectious disease tests (MIDTs). We included 345 patients with International Classification of Diseases (ICD)-10 codes for transplant (Z940-Z942, Z944, Z9481, Z9483, Z9484) with at least two tests (within 7 days) in January 2019 and at least one test in December 2020 to find patients in the post-transplant period. We analyzed two cohorts: kidney transplant recipients (KTRs; 40%) and non-KTR (60%) then followed them longitudinally for the study period. In KTR cohort, high-to-low proportion of ordered MIDT was blood BK virus (bBKV) followed by cytomegalovirus (CMV); in non-KTR cohort, CMV was followed by Epstein–Barr virus (EBV). KTR cohort positivity was highest for urine BK virus (uBKV; 58%) followed by EBV (46%), bBKV (40%), and CMV (31%). Non-KTR cohort positivity was highest for uBKV (64%), EBV (51%), CMV (30%), bBKV (8%), and adenovirus (7%). All patients were tested at progressively longer intervals from the date of the first post-transplant ICD-10-coded test. More than 40% of the KTR cohort were tested less frequently for EBV and bBKV, and more than 20% of the non-KTR cohort were tested for EBV less frequently than published guidelines 4 months after transplant. Despite regular testing, the results of MIDT testing for KTR and non-KTR patients in the post-transplant period are not aligned with published guidelines.

**IMPORTANCE:**

Guidance for post-transplant infectious disease testing is established, however, for certain infections it allows for clinician discretion. This leads to transplant center policies developing their own testing/surveillance strategies based on their specific transplant patient population (kidney, stem cell, etc.). The Organ Procurement and Transplant Network (OPTN) has developed a strategic plan to improve and standardize the transplant process in the US to improve outcomes of living donors and recipients. Publishing national reference lab data on the testing frequency and its alignment with the recommended guidelines for post-transplant infectious diseases can inform patient uptake and compliance for these strategic OPTN efforts.

## INTRODUCTION

In the US, more than 39,000 organ transplants were performed in 2020. Kidney transplants (KTs) were the most common, followed by liver, heart, lung, and others, including dual organ, pancreatic, and intestinal transplantation (non-KT) (1). Post-transplant infections (PTI) are still a significant risk to successful outcomes for both solid organ transplant (SOT) and human stem cell transplant (HSCT) recipients.

Donors and recipients undergo pre-transplant screening, in order to select a suitable donor, appropriate prophylaxis, and management in the post-transplant period. Immuno-suppressive regimens are standardized; therefore, the most common infections occur in a relatively predictable pattern depending on the time elapsed since transplantation (2, 3). Factors influencing PTI include transplant type, immunosuppressive regimens, subclinical viral infections, graft dysfunction, or epidemiologic exposures (e.g., travel or food). Post-transplant testing guidelines were established to monitor and guide therapeutic interventions in transplant recipients. We hypothesize that there are gaps in adherence to the recommended frequency of laboratory testing in post-transplant patients. We undertook this study to identify the most common viral PTIs and assess their positivity rates and alignment of frequency of testing with published guidelines among SOT recipients.

## MATERIALS AND METHODS

In this retrospective analysis of Quest Diagnostics data, we included patients of all ages with International Classification of Diseases, Revision 10 (ICD-10) codes for transplant (Z940-Z942, Z944, Z9481, Z9483, Z9484) who had at least two tests (within 7 days) in January 2019 and at least one test since December 2020. This process provided a relatively homogeneous group of patients who are more likely to have started the post-transplant testing in January 2019 and had a complete set of testing records in our laboratory. We divided this group into kidney transplant recipients (KTRs) and non-KTR cohorts and followed up longitudinally with each throughout the study period.

We studied molecular infectious disease tests (MIDTs) ordered longitudinally for the study patients. The assays were performed per the standard operating procedures, and all MIDTs were quantitative polymerase chain reaction (PCR) assays. We studied the volume and positivity rates to understand the landscape of PTIs for adenovirus, BK virus (BKV; blood and urine), cytomegalovirus (CMV), and Epstein–Barr virus (EBV). However, to evaluate frequency of testing and its alignment to the guidelines, we focused on BKV, CMV, and EBV only.

We estimated frequency of testing for BKV, CMV, and EBV using test orders for over 12 months after the post-transplant period. To study the alignment of frequency of CMV and EBV, we incorporated recommendations for clinical visits of patients post-transplant by the Renal Association (4). The frequency intervals recommended: 2–3 times weekly in the first month after transplantation; 1–2 times weekly for months 2–3; every 2–4 weeks for months 4–6; every 4–6 weeks for months 6–12; and every 3–6 months after that. For EBV, it is recommended to follow monthly for 6 months, and 3 monthly to 1 year. While guidelines exist for post-transplant CMV (5) and EBV (6) for SOT (including non-KTR) recipients, the Renal Association framework for post-transplant clinical visits was most specific and well defined. Therefore, the Renal Association guidelines for post-transplant clinical visits were used in the present study for the comparison of KTR and non-KTR testing frequency. In the context of the present study, testing dates represented a clinical visit. For the BKV testing frequency alignment, we incorporated recommendations for testing frequencies from the American Society of Transplantation Infectious Diseases Community of Practice (AST ID COP) (3), which outlined intervals of once a month for the first 9 months, then every 3 months for 9–24 months after transplant.

Using the statistical programming software R (version 3.6.1), we evaluated the statistical significance of comparisons using the 95% CI or the proportion *Z*-test. Kruskal–Wallis statistical analysis was used to test the PTI testing frequency distribution difference between KTR and non-KTR patients.

## RESULTS

Among 345 patients in our study, 137 (40%) of patients were categorized as KTR while the remaining 60% were categorized as non-KTR. In the non-KTR group, 21% were lung transplants, 13% were liver transplants, and other types of transplants (such as dual organs, heart, stem cells, and bone marrow) ranged from 1% to 5%. (Table S1). The highest number of patients tested was for CMV (*n* = 294), followed by blood BK virus (bBKV) (*n* = 149), EBV (*n* = 104), urine BK virus (uBKV) (*n* = 71), and adenovirus (*n* = 37). Of the 12,115 MIDTs, 6,248 (52%) were categorized as KTR and 5,867 (48%) were categorized as non-KTR. Overall, MIDTs were ordered in the following proportions: CMV (56%), bBKV (25%), EBV (10%), uBKV (7%), and adenovirus (1%).

The pattern of MIDTs ordered for KTR and non-KTR was different; their positivity rates were also different. In the KTR cohort, bBKV was most frequently ordered (*n* = 2,887 with 19% positivity), followed by CMV (*n* = 2,110 with 10% positivity), uBKV (*n* = 776 with 52% positivity), EBV (*n* = 462 with 49% positivity), and adenovirus (*n* = 13 with no positivity) (Table 1). For the non-KTR cohort, CMV was the most frequently ordered (*n* = 4,716 with 6% positivity), followed by EBV (*n* = 789 with 35% positivity), adenovirus (*n* = 147 with 3% positivity), bBKV (*n* = 118 with 2% positivity), and uBKV (*n* = 97 with 54% positivity) (Table 1).

**TABLE 1:** MIDTs on the study population (345) from January 2019 to December 2020^*a*^

Test group	Age, Mean	Results, *N*	Volume, %	Positive results, *N*	Positive results, Rate (%)
ALL					
CMV DNA	57.1	6,826	56	465	6.8
BKV DNA	56.3	3,005	25	552	18.4
EBV DNA	50.5	1,251	10	499	39.9
uBKV DNA	53.9	873	7	454	52.0
Adenovirus DNA	59.0	160	1.3	4	2.5
Parvovirus DNA	56.8	14	0.12	0	0.0
Herpes virus 6 DNA	53.4	13	0.11	0	0.0
Aspergillus DNA	51.2	6	0.05	0	0.0
JC virus DNA	59.0	3	0.02	0	0.0
*Pneumocystis jiroveci*	10.3	2	0.02	0	0.0
Total		12,153			
KTR Cohort					
BKV DNA	56.2	2,887	46	549	19.0
CMV DNA	54.5	2,110	34	202	9.6
uBKV DNA	53.2	776	12	402	51.8
EBV DNA	56.6	462	7	225	48.7
Adenovirus DNA	59.7	13	0.21	0	0.0
Parvovirus DNA	44.2	3	0.05	0	0.0
JC virus DNA	56.7	2	0.03	0	0.0
Total		6,253			
non-KTR Cohort					
CMV DNA	58.2	4,716	80	263	6
EBV DNA	46.9	789	13	274	35
Adenovirus DNA	58.9	147	2	4	3
BKV DNA	59.2	118	2	3	3
uBKV DNA	59.6	97	1.6	52	54
Parvovirus DNA	60.2	11	0.2	0	0
Herpes virus 6 DNA	53.4	13	0.2	0	0
Aspergillus DNA	51.2	6	0.10	0	0
JC virus DNA	63.6	1	0.02	0	0
*Pneumocystis jiroveci*	10.3	2	0.03	0	0
Total		5,900			

^*a*^ JC-John cunningham; UNOS-United Network for Organ Sharing.

**FIG 1:**
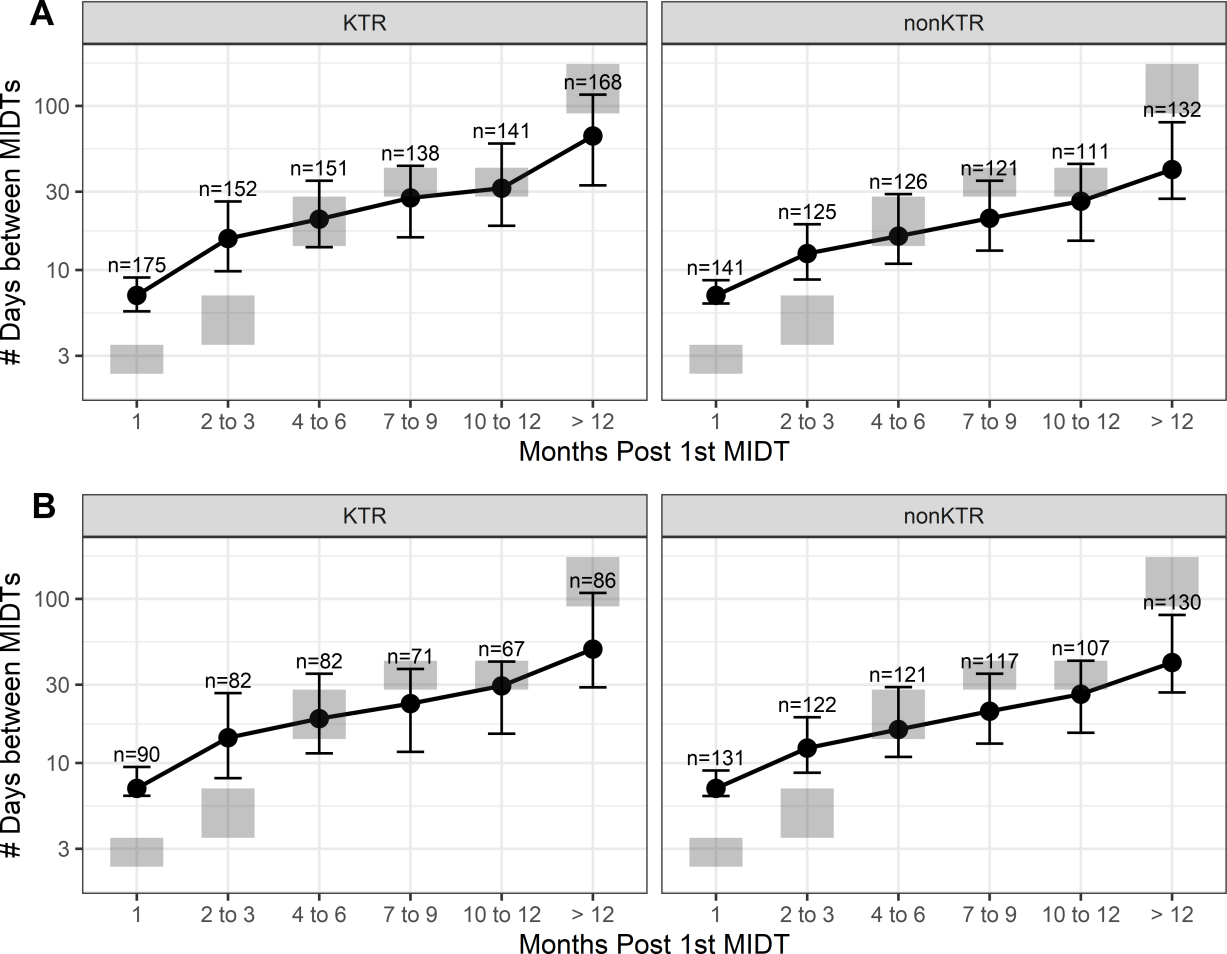
(A) The average number of days between testing for any MIDT over time for KTR and non-KTR. (B) The average number of days between testing for CMV over time for both KTR and non-KTR.

The average number of days between MIDT orders increased over time, a pattern that is consistent with the Renal Association guideline (4) for KTR, and we observed a similar pattern for non-KTR cohorts (Fig. 1A and B). We observed MIDT order frequency of mean 7 days for both KTR and non-KTR cohorts within the first month from the first order in our database. However, the distributions of MIDT frequency for KTR and non-KTR cohorts were statistically different for months 2–12 (*P* < 0.005). The mean days of testing frequency were wider in the non-KTR cohort than in the KTR cohort (Table 2).

**TABLE 2:** Guidelines for the frequency of testing

		Testing interval (days) used in this study					
Pathogen	Frequency of testing post-transplant	Month 1	Month 2–3	Month 4–6	Month 7–9	Month 10–12	>12 Months
CMV (4)	2–3 times weekly in the first month 1–2 times weekly for months 2–3 Every 2–4 weeks for months 4–6 Every 4–6 weeks for months 6–12 Every 3–6 months after that	3.5	7	28	42	42	180
EBV (4)	Measured immediately after transplantation Monthly for 6 months 3 monthly for 7–1 year	30	30	30	90	90	90
BKV (3)	Once a month for the first 9 months Every 3 months for 9–24 months	30	30	30	30	90	90

The Renal Association provides guidelines concerning the frequency of clinical visits only; for CMV monitoring, it is left to the institute-specific protocols (4). Therefore, we compared clinical visit frequency in Renal Association guidelines to CMV testing frequency in this data set. In both KTR and non-KTR cohorts, the orders for CMV testing were less frequent for months 1–3 than the clinical visit interval mentioned in the Renal Association guidelines. However, CMV test orders were aligned with the published clinical visit frequency guidelines for months 4–12, and more frequently ordered than the guidelines after 12 months. (Fig. 1B andTable 2).

**FIG 2:**
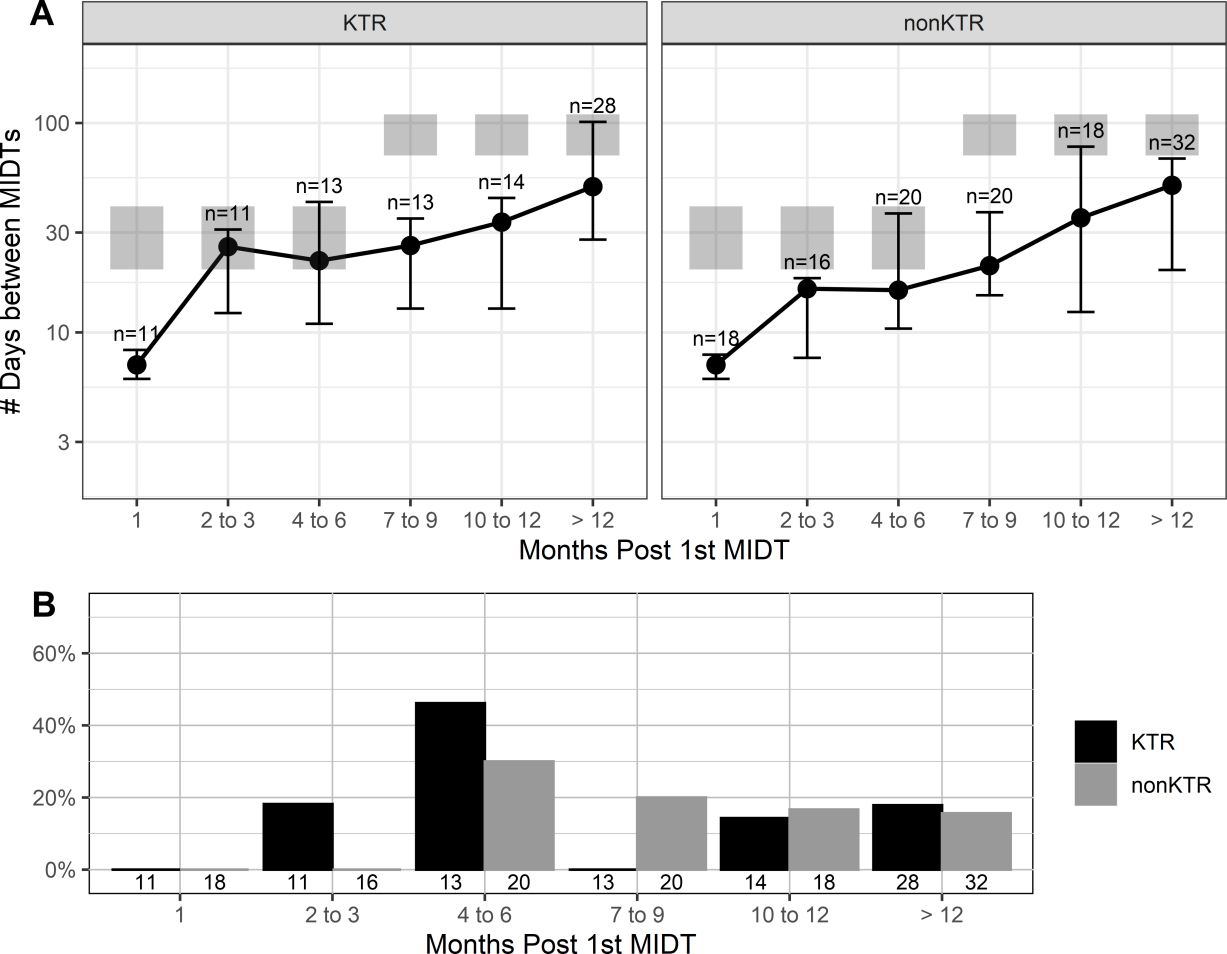
(A) The average number of days between testing for EBV in KTR and non-KTR. (B) Percent patients tested less frequently for EBV than the recommended frequency of EBV testing guidelines over time for both KTR and non-KTR cohorts.

EBV testing was ordered less frequently than CMV in both KTR (7% EBV vs. 34% CMV) and non-KTR (13% EBV vs. 80% CMV) cohorts. Compared to the clinical visit guidelines (4), EBV tests were ordered more frequently in the KTR and non-KTR cohort for all periods except months 2–6 in our data set (Fig. 2A). Though mean testing frequency aligned with testing guidelines in months 4–6, >40% of the KTR and >20% of the non-KTR cohort were ordered less frequently for EBV than the recommended frequency of EBV testing in the guidelines (Fig. 2B) (4).

**FIG 3:**
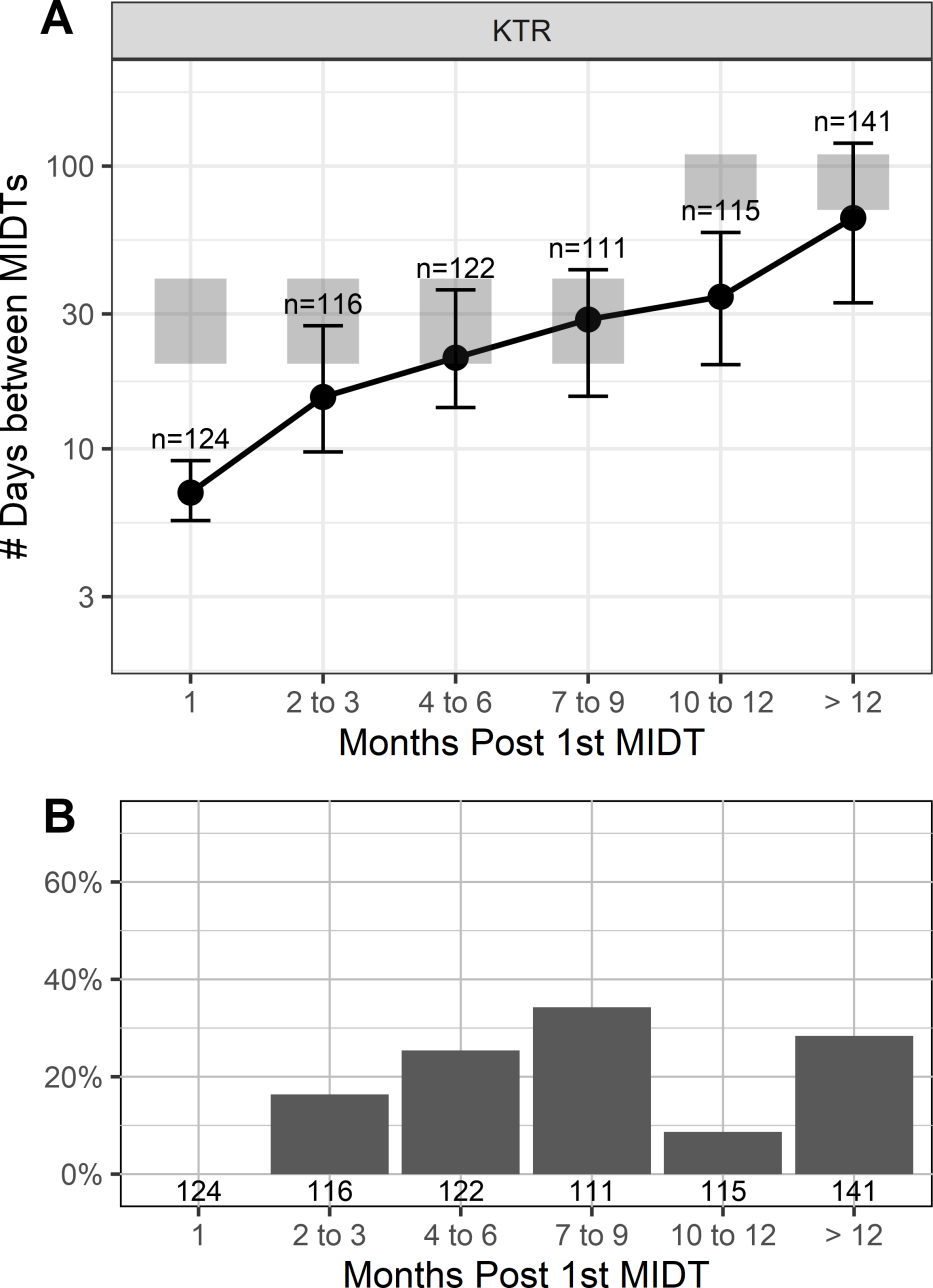
(A) The average number of days between testing for BKV in KTR cohort. (B) Percent patients tested less frequently for BKV than the recommended frequency of BKV testing guidelines over time for KTR cohort.

Compared to BKV testing guidelines (3), bBKV test orders were more frequent in the KTR cohort for months 1–3 and were similarly frequent for months 7–12+ while monitoring this data set (Fig. 3A). Though mean test frequency aligned similarly to testing guidelines for months 7–12+, >40% of the KTR cohort were tested less frequently than bBKV testing guidelines (Fig. 3B) (3).

## DISCUSSION

Taking into account the ICD coding in this data set, the top five organ transplants proportionately match that of the top five organ transplant listed by UNOS’ top five: kidney, lung, liver, kidney/pancreas, and heart (1). This is important and affirming as an initial survey/publication of national reference lab data for testing during post-transplant infectious disease period.

More frequent testing for bBKV for the KTR cohort is understandable as BKV is the primary cause of polyomavirus‐associated nephropathy after KT (3). Currently pre-transplant screening for either bBKV or uBKV is not recommended for organ allocation. Therefore, the AST ID COP recommends screening for BKV‐DNAemia for KT recipients monthly until month 9, and then every 3 months until 2 years after transplant (3, 4). Excretion of BKV occurs in the urine more often than presence of BKV in the blood in KTRs. We observed a similar pattern: the positivity rate for uBKV samples was higher (52%) compared to the positive rate in the blood (18%). As >40% of the KTR cohort were tested less frequently than bBKV testing guidelines, this is an area worthy of testing stewardship consideration to improve patient compliance to guidelines. Of note, bBKV positivity has a positive predictive value of 30–50% for BK-associated nephropathy with a window period of 2–8 weeks (3). The sooner a bBKV positive patient can undergo preemptive reduction in immunosuppression, the better chance of avoiding progressive stages of BK-associated nephropathy and increased likelihood of irreversible damage, fibrosis, tubular atrophy, and premature graft failure (3).

Despite effective antiviral prophylaxis and therapies, CMV remains an important pathogen in transplant recipients (5, 7, 8). We observed the test orders for CMV were highest in the non-KTR cohort and second highest in KTR cohort. Even though symptomatic CMV infections are rare during effective antiviral prophylaxis, after the cessation of prophylaxis, 25–40% of patients develop symptomatic disease (7). Therefore, monitoring CMV in transplant patients is critical for managing patient prophylaxis.

EBV serologic testing before transplantation is recommended for all donors and recipients (6). EBV seronegative recipients are at higher risk for primary EBV infection, associated with a significantly increased risk of post-transplant lymphoproliferative disorder (PTLD) (6, 9). The seroprevalence of EBV in the US is 83% by 19 years of age (10). Therefore, PTLD occurs in up to 20% of pediatric organ recipients and <1% of adults. All high-risk patients (EBV serology: donor-positive; recipient-negative) are screened using quantitative EBV molecular assays immediately after transplantation, monthly for 6 months, and every 3 months for up to 1 year (11–13). We observed 20–40% less frequent EBV testing compared to the recommended frequency. However, the positivity rates of EBV samples were high in both cohorts (KTR, 49%; non-KTR, 35%). As this data set is confined to lab results and does not include clinical data, there is no way of knowing the index of clinical suspicion for EBV/PTLD related to EBV clinical visits and/or testing frequency (14, 15). One possible explanation is that the clinicians are testing for EBV only when they have high index of clinical suspicion of EBV; then due to less frequent testing, more patients reach viremic state. The high viremic patients are candidates for reducing immunosuppression; failure to clear viremia raises concerns for PTLD (14, 15).

Patients were tested for MIDT at progressively longer intervals over time with the greatest similarity to guidelines in months 4–12 after first orders in our database for CMV and months 7–12 for EBV. BKV testing occurred more frequently than guidelines for all KTRs throughout the entire study period. The difference between frequency of MIDT orders for KTR and non-KTR cohorts showed statistical difference for all post-transplant phases (*P* < 0.005) except for the first month after transplant (*P* = 0.66).

There are a few limitations of this study: (1) a selection bias for this national reference lab data set may exist as most test samples are collected on ambulatory individuals at (out)patient service centers (PSCs) across the US; (2) there are no clinical data (even for a small subset) to inform testing in proximity to the date of transplant, nor knowing whether a given test/result was donor- or recipient-derived; (3) for evaluation of frequency of testing, we used recommended frequency for clinical visits as an analogue in the absence of specific CMV testing frequency guidelines.

### Conclusion

We observed CMV, bBKV, and EBV tests, as the most frequently ordered MIDT in our cohort of KTRs and non-KTRs. Frequency of MIDT test orders progressively widened over time as recommended in the guidelines. Of note, >40% of KTR and >20% of non-KTR cohorts were tested less frequently than guidelines suggest. These data from a national reference laboratory demonstrate that gaps occur in adhering to recommended frequency of testing, with viral infections of greatest concern. Post-transplant MIDT orders for KTR and non-KTR patients are different and despite regular testing, there is a gap in testing the patients. In the absence of clinical data, we suggest that larger prospective studies may be done to confirm our findings from this retrospective data analysis.

## Data Availability

De-identified data will be provided upon request.

## SUPPLEMENTAL MATERIAL

Supplemental material spectrum.03575-23-s0001.jpg Visual abstract.

Supplemental tables spectrum.03575-23-s0002.xlsx Tables S1 and S2.

ASM does not own the copyrights to Supplemental Material that may be linked to, or accessed through, an article. The authors have granted ASM a non-exclusive, world-wide license to publish the Supplemental Material files. Please contact the corresponding author directly for reuse.
