# Supplementary figures and images for: Burden and frequency of viral testing of kidney and non-kidney transplant recipients
Source: Microbiol Spectr. 2024 May 6;12(6):e03575-23. doi: 10.1128/spectrum.03575-23 (PMC11237713; doi:10.1128/spectrum.03575-23)

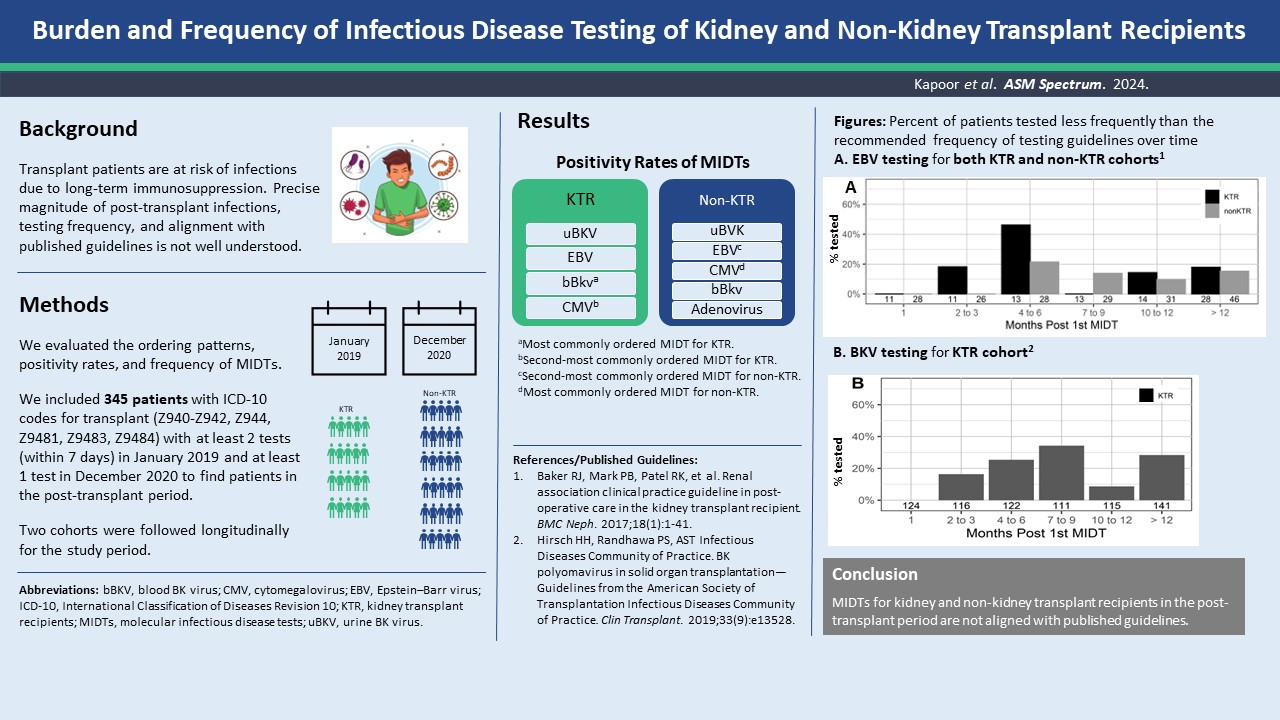

Supplement: Supplemental material — Visual abstract. [file spectrum.03575-23-s0001.jpg]
